# Selection of different surgical methods for uterine fibroids: Protocol for a retrospective clinical study

**DOI:** 10.1097/MD.0000000000028378

**Published:** 2021-12-23

**Authors:** Ya-Hong Liu, Yu-Hong Qiu, Ya Ru, Yan-Qing Liu, Dan Wang, Ping-An Zhang

**Affiliations:** The Second Affiliated Hospital of Shaanxi University of Chinese Medicine, Xianyang, Shaanxi, P.R. China.

**Keywords:** clinical trial, myomectomy, protocol, uterine fibroids

## Abstract

**Introduction:**

Uterine fibroids is a common benign tumor disease of the female reproductive system. The main methods of current clinical treatment of uterine fibroids are conservative treatment and surgical treatment. With the rise of the concept of minimally invasive surgery in gynecology, laparoscopic myomectomy, and vaginal myomectomy have been widely used.

**Methods/design:**

This study plans to retrospectively analyze 150 patients with uterine fibroids. They will be divided into laparoscopic myomectomy, vaginal myomectomy group, and open hysteromyoma resection group. This study will compare the intraoperative blood loss, operation time, postoperative exhaust time, postoperative hospital stay and postoperative complications of different surgical methods.

**Discussion:**

This study will compare the clinical efficacy of these 3 common surgical methods through retrospective medical record analysis, and provide more reliable evidence-based medical evidence for clinical treatment choices.

## 1 Introduction

In recent years, the incidence of uterine fibroids has been on the rise. Myomectomy is an ideal surgical procedure to preserve female fertility.^[1]^ Uterine fibroids are common benign tumor diseases of the female reproductive system, which mostly occur in young and middle-aged women. The clinical incidence of this disease can reach 20%.^[2]^ Uterine fibroids can easily lead to uterine enlargement, menstrual disorders, vaginal bleeding, etc, and often cause infertility in women during reproductive periods. In addition, long-term vaginal bleeding easily induces secondary anemia, which affects the patient's quality of life and life safety. At present, the main methods of clinical treatment of uterine fibroids are conservative treatment and surgical treatment. With the rise of minimally invasive concepts in gynecological surgery, laparoscopic myomectomy and vaginal myomectomy have been widely used. Laparoscopic myomectomy has the advantages of low invasiveness, high curative effect, and high aesthetics,^[3]^ and is widely used in clinical practice. Nowadays, vaginal myomectomy is a newly emerged gynecological minimally invasive surgery method,^[4]^ due to its short operation time and intraoperative bleeding The advantage of small amount is a common minimally invasive procedure of myomectomy. Although the traditional open hysteromyoma resection has a large abdominal wall incision, slow recovery of the body, and a longer hospital stay, it still has an irreplaceable position in surgical procedures with larger tumors and longer gestational weeks. This study will compare and analyze the clinical effects of the above 3 surgical methods, and explore practical and effective clinical surgical programs.

## 2 Methods/design

### 2.1 Study design and settings

**Figure 1:**
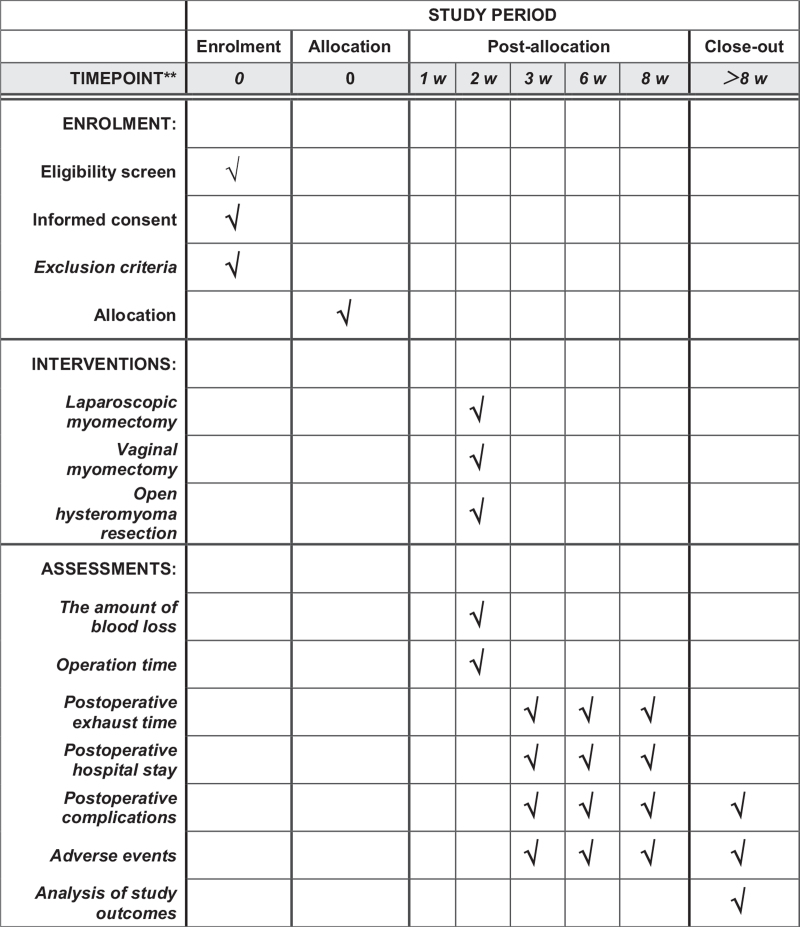
SPIRIT figure for the schedule of enrollment, interventions, and assessments. SPIRIT = Standard Protocol Items: Recommendations for Interventional Trials.

This study is a retrospective clinical trial. At present, we have completed the pre-registration (ChiCTR2100053314) at the China Clinical Trial Center. The time points for registration and evaluation are shown in the standard protocol project: Intervention Trial Recommendation chart (Fig. 1).

### 2.2 Participants

This study will select patients who underwent myomectomy in the Second Affiliated Hospital of Shaanxi University of Chinese Medicine in the past 5 years. We will divide these patients into 3 groups according to different surgical methods. Laparoscopic myomectomy group, vaginal myomectomy group, open hysteromyoma resection group, 50 cases in each group. All patients were diagnosed with benign cervical lesions, and there were no contraindications to surgery.

#### 2.2.1 Diagnostic criteria

Common clinical symptoms of uterine fibroids include increased menstrual flow and prolonged menstrual periods, larger fibroids, palpable masses in the lower abdomen, increased leucorrhea, compression symptoms (including compression of the bladder, rectum, and ureters), and other symptoms (lower abdominal pain), Back pain), etc; Gynecological examination: the uterus is enlarged and the surface is uneven with single or multiple nodular protrusions; Auxiliary examination: vaginal ultrasound supports the diagnosis of uterine fibroids; Postoperative disease Physical examination is the gold standard for diagnosis: the pathological section after resection confirms the diagnosis of uterine fibroids. Diagnosed as uterine fibroids through the above symptoms, signs and auxiliary examinations.

#### 2.2.2 Eligibility criteria

- Female patients between 40 to 50 years old;
- The uterus enlarges to the size of 8 to 20 weeks of pregnancy;
- There is no contraindication to surgery, and the operation method is Laparoscopic myomectomy or Vaginal myomectomy or open hysteromyoma resection;
- All surgeons are doctors with rich clinical experience at the level of deputy chief physician or above;
- Complete clinical data;

The exclusion criteria are as follows:

- Past pelvic and abdominal surgery history;
- Severe pelvic adhesions;
- Combined diseases that significantly affect surgery and prognosis, such as diabetes, hypertension, heart disease, liver, kidney, and other important organ complications;
- With a history of gynecological malignant tumors or other tumors;
- History of blood disease;
- History of taking anticoagulant drugs;
- Severe vaginal inflammation;
- Combined with other non-pelvic surgery cases, such as appendectomy, etc;
- Laparoscopy and vaginal hysterectomy were converted to open surgery.

### 2.3 Interventions

Use iodophor cotton ball to sterilize the vagina for 3 days, 2 times a day before surgery; clean the enema the night before surgery and fast water for at least 8 hours; general anesthesia for laparoscopic myomectomy, combined spinal-epidural anesthesia or general anesthesia for vaginal myomectomy and open hysteromyoma resection.

- Laparoscopic myomectomy group: take the bladder lithotomy position, insert a pneumoperitoneum needle just above the umbilical edge, insert a 10 mm umbilical puncture cannula, insert a laparoscope, CO_2_ pneumoperitoneum, pneumoperitoneum pressure 12 to 13 mm Hg, after the pneumoperitoneum is completed, Routine 3-point puncture in the abdomen, puncture cannula diameter 5 mm, uterine body injection of oxytocin, according to the size, location, direction of the uterine fibroids, cut the surface membrane of the fibroids, separate the tumor body, while using electrocoagulation to stop bleeding. The muscular layer of the tumor cavity was sutured first, and then the seromuscular layer was sutured, and the drainage tube was indwelled for 24 hours after the operation.
- Vaginal myomectomy group: take the bladder lithotomy position, choose the vaginal vault incision according to the tumor position, cut the vaginal mucosa, advance to the front and back of the uterus, incise the peritoneum, and expose the uterus, cut the surface membrane of the fibroids, and separate the tumor body, if the fibroids are too large, the tumors will be removed after fragmentation. Routine stitching after removal.
- Open hysteromyoma resection group: According to the routine open myomectomy operation.

### 2.4 Outcomes

The choice of outcome indicators will be evaluated according to the amount of blood loss, operation time, postoperative exhaust time, postoperative hospital stay and postoperative complications. In addition, we will calculate the recurrence rate of uterine fibroids through follow-up.

### 2.5 Statistical analysis

Data management uses EXCEL software to build a database, double entry, check for outstanding values, and lock. Statistical analysis will be performed using SPSS 25.0 software for statistical analysis. The normality of the measurement data is tested. The data obeying the normal distribution is Student *t* test, which is expressed by mean ± standard deviation. The data not obeying the normal distribution is rank sum test. And marginal homogeneity test; count data are expressed by rate and composition ratio, and comparison is performed by chi-square test; repeated measurement data are expressed by mean ± standard deviation, intra-group comparison is performed by analysis of variance of repeated measurement data, and inter-group comparison is by multivariate analysis of variance. *P* ≤ .05 indicates that the difference is statistically significant.

### 2.6 Data management

Information obtained from the evaluation of each participant will be recorded on a paper print-out. The information will then be handwritten on a paper document case report form and entered into an Excel file for future statistical analyses. In accordance with the Personal Information Protection Act, the names of all participants will not be disclosed, and a unique identifier number given during the trial will be used to identify participants. All of the participants will be informed that the clinical data obtained in the trial will be stored in a computer and will be handled with confidentiality. The participants’ written consent will be stored by the principal investigator.

### 2.7 Ethics

The study will be conducted under the Declaration of Helsinki principles, as well as following the norms of good clinical practice. Recruitment of patients has not started in this study. The study plan will be submitted to the ethics committee of the Second Affiliated Hospital of Shaanxi University of Traditional Chinese Medicine for review. The final study version will be approved by the Ethics Committee of the Second Affiliated Hospital of Shaanxi University of Chinese Medicine. We will not include subjects without the approval of the ethics committee.

## 3 Discussion

Uterine fibroids is one of the common benign tumors in female gynecology, with a higher incidence in women aged 30 to 50. The cause of uterine fibroids is currently unclear, and may be affected by estrogen and progesterone in the receptor.^[5]^ Common symptoms include uterine bleeding, mass and compression symptoms in the abdomen, and a few painful phenomena. Some uterine fibroids may have no symptoms. Common treatments include medications such as mifepristone, uterine artery embolization and surgical treatment, of which surgical treatment is the main one. However, myomectomy is widely used as an ideal surgical method for preserving female fertility. At the same time, this surgical method has little effect on the patient's hypothalamic-pituitary-uterine axis, which is beneficial to the patient's postoperative recovery. Since 1844, the first successful implementation of Open hysteromyoma resection, due to the corresponding complications, this surgical method was not widely used until the middle of the 20th century.^[6]^ Open hysteromyoma resection is now relatively mature. With the development of the concept of minimally invasive, laparoscopic myomectomy and vaginal myomectomy are now widely accepted, and gradually replace open hysteromyoma resection.

Laparoscopic myomectomy has the advantages of small incision, less postoperative pain, less interference to the abdominal cavity, faster postoperative recovery, and shorter hospital stay. At the same time, its pelvic vision is clear, which is conducive to comprehensive observation of abdominal organs and pelvic adhesions. However, the operation is complicated, and it is suitable for uterine fibroids patients with fibroids ≥4 cm in diameter but ≤10 cm in single or multiple (<4). Uterine fibroids with a diameter of more than 10 cm or more than 4 uterine fibroids are considered to be contraindications for laparoscopic surgery,^[7]^ which may increase the amount of intraoperative bleeding and increase the difficulty of suture. Since fibroids cannot be directly touched, the experience and skills of the surgeon have a great influence on the results of the operation, and fibroids with smaller diameters are likely to be missed, resulting in recurrence of the disease. In addition to the advantages of laparoscopic surgery, Vaginal myomectomy compresses the blood vessels of the uterus due to the extreme flexion or flexion of the uterus, which reduces the amount of bleeding and makes up for the shortcomings of laparoscopic surgery. At the same time, vaginal myomectomy takes advantage of the feature that the uterus and vagina are connected to the outside world, and there is no scar on the abdominal wall after the operation to relieve the pain of the patient.

Vaginal myomectomy is easy to cause postoperative hematoma, the formation rate is 11%, and the open-abdominal type is only 8%.^[8,9]^ The 3 surgical methods analyzed in this project have their own points and shortcomings. Open hysteromyoma resection has the widest range of application, with fewer contraindications, and is irreplaceable clinically. However, the amount of bleeding during open hysteromyoma resection is higher than other surgical methods. Laparoscopic myomectomy has many advantages, but the operation is complicated and the operation time is longer. Vaginal myomectomy has the advantages of the laparoscopic group, its intraoperative blood loss was significantly lower than that of the laparoscopic group, and the operation time was also less than that of the laparoscopic group. Vaginal myomectomy is more advantageous than the open and laparoscopic groups in its indications. However, the number of fibroids is generally not more than 2. For larger fibroids, it needs to be minced and then removed, which also increases the risk of intraoperative bleeding. At the same time, with the improvement of technology and equipment, laparoscopic assisted vaginal myomectomy combines the advantages of both, and at the same time expands the indications of laparoscopic myomectomy, providing more options for replacing open abdominal. This study intends to compare the clinical efficacy of these 3 common surgical methods through retrospective medical record analysis, and to provide more reliable evidence-based medical evidence for clinical treatment choices.

## Acknowledgments

The authors would like to thank all the trial participants. The authors are grateful for the support for this study: trial coordinating team, surgical staff, nurses, and research departments.

## Author contributions

**Formal analysis:** Ya-Hong Liu.

**Investigation:** Yu-Hong Qiu.

**Methodology:** Ya Ru.

**Supervision:** Yan-Qing Liu, Dan Wang.

**Writing – original draft:** Ping-An Zhang.

**Writing – review & editing:** Ping-An Zhang.
